# Rapid Genomic Diagnosis of Fungal Infections in the Age of Next-Generation Sequencing

**DOI:** 10.3390/jof7080636

**Published:** 2021-08-05

**Authors:** Chi-Ching Tsang, Jade L. L. Teng, Susanna K. P. Lau, Patrick C. Y. Woo

**Affiliations:** Department of Microbiology, Li Ka Shing Faculty of Medicine, The University of Hong Kong, Pokfulam, Hong Kong, China; microbioct@connect.hku.hk (C.-C.T.); llteng@hku.hk (J.L.L.T.)

**Keywords:** genomic diagnosis, next-generation sequencing, NGS, fungal infection, mycosis

## Abstract

Next-generation sequencing (NGS) technologies have recently developed beyond the research realm and started to mature into clinical applications. Here, we review the current use of NGS for laboratory diagnosis of fungal infections. Since the first reported case in 2014, >300 cases of fungal infections diagnosed by NGS were described. *Pneumocystis jirovecii* is the predominant fungus reported, constituting ~25% of the fungi detected. In ~12.5% of the cases, more than one fungus was detected by NGS. For *P. jirovecii* infections diagnosed by NGS, all 91 patients suffered from pneumonia and only 1 was HIV-positive. This is very different from the general epidemiology of *P. jirovecii* infections, of which HIV infection is the most important risk factor. The epidemiology of *Talaromyces marneffei* infection diagnosed by NGS is also different from its general epidemiology, in that only 3/11 patients were HIV-positive. The major advantage of using NGS for laboratory diagnosis is that it can pick up all pathogens, particularly when initial microbiological investigations are unfruitful. When the cost of NGS is further reduced, expertise more widely available and other obstacles overcome, NGS would be a useful tool for laboratory diagnosis of fungal infections, particularly for difficult-to-grow fungi and cases with low fungal loads.

## 1. Introduction

Traditionally, laboratory diagnosis of fungal infections relies on isolation of the fungal pathogen and/or direct microscopic examination of the clinical specimens with the help of stains. Culturing fungal pathogens, particularly filamentous fungi, usually requires 1–2 weeks of incubation. Even if isolation of the fungus is successful, identification is sometimes very challenging and requires expertise, which may not be routinely available in clinical microbiology laboratories. As for staining and microscopy, it is neither sensitive nor specific [1,2,3,4], although if positive can give a clue to early commencement of antifungal agents. Hence, antigen and antibody detection have been increasingly used, aiming at more rapid and specific diagnosis. The most sensitive, specific and widely available antigen or antibody detection test for fungal infection is the cryptococcal antigen assay [4]. However, for most other antigen or antibody detection assays, they suffer from major drawbacks such as being non-specific (e.g., β-d-glucan detection) [2,5], insensitive (e.g., galactomannan antigen and antibody detection) [2,5], and/or only in-house available (e.g., *Talaromyces marneffei* and *Aspergillus fumigatus* antigen and antibody detection) [6,7,8,9,10,11].

In the recent few decades, DNA amplification and sequencing have been increasingly used for laboratory diagnosis of fungal infections [12]. Fungus-specific primers for PCR and quantitative real-time PCR amplification has been used for diagnosis of *Aspergillus*, *Candida, Mucorales,* and *Pneumocystis jirovecii* infections [13]. Detection of antifungal resistance is also possible through amplification of resistance genes and picking up mutations, such as TR_34_/L98H in *Cyp51A* in azole-resistant *A. fumigatus* strains [14,15]. In the last 10 years, pan-fungal PCR assays targeting DNA markers such as the internal transcribed spacer region (ITS) and 28S nrDNA (D1/D2 region) followed by Sanger sequencing have been widely used for both identification of fungi isolated as well as detection of fungal pathogens directly from clinical specimens [13]. Since the PCR primers are designed in such a way that they will anneal to and amplify conserved target regions, theoretically they are able to pick up and identify any fungus in/from a clinical sample. In addition to being able to find known pathogens, this technology has also played a pivotal role in the discovery of previously undescribed fungal pathogens [16,17,18,19,20].

In the recent few years, next-generation sequencing (NGS) technologies have developed beyond the research realm and started to mature into clinical applications. The initial use of NGS was mainly for genome sequencing, complementing and then replacing the traditional genome sequencing approach which involved cloning of DNA fragments, sequencing of individual clones by Sanger sequencing and genome walking, and assembly of the sequenced clones. With the advancement of sequencing chemistries, computer hardware and software as well as computational capacity, new NGS platforms and versions have been generated on an exponential scale. Different NGS platforms have their own advantages and disadvantages. Short-read sequencers such as Illumina, MGI’s DNBSEQ and Ion Torrent platforms are well-known for their low error rates and sequencing costs per base. Different sequencer models are available from these platforms, which serve a multitude of purposes and sequencing throughputs on all scales, ranging from high throughput (e.g., Illumina’s HiSeq System, up to 600 Gb) to benchtop and low throughput (e.g., Illumina’s MiSeq System, up to 15 Gb), with a maximum read length of 400 bp. More recently, long-read sequencers such as Pacific Biosciences (PacBio) and Oxford Nanopore Technologies platforms have been developed and they can generate long reads of ≥50 kb, which facilitates *de novo* genome assembly and resolution of repetitive genomic regions. However, PacBio platforms are known to possess a higher input DNA requirement (i.e., quantity in micrograms) and lower read accuracy compared with other NGS platforms [21,22]. The recent invention of Oxford Nanopore Techonologies’ MinION device has expedited the use of NGS in laboratory diagnosis, due to its low equipment cost, short turn-around-time and portable size. In this article, we review the current use of NGS in laboratory diagnosis of fungal infections. The opportunities, obstacles, and future trend of using NGS are also discussed. However, details of the various NGS technologies, which have been reviewed in other articles [23,24,25], will not be covered.

## 2. Patient Selection and Procedure

**Figure 1:**
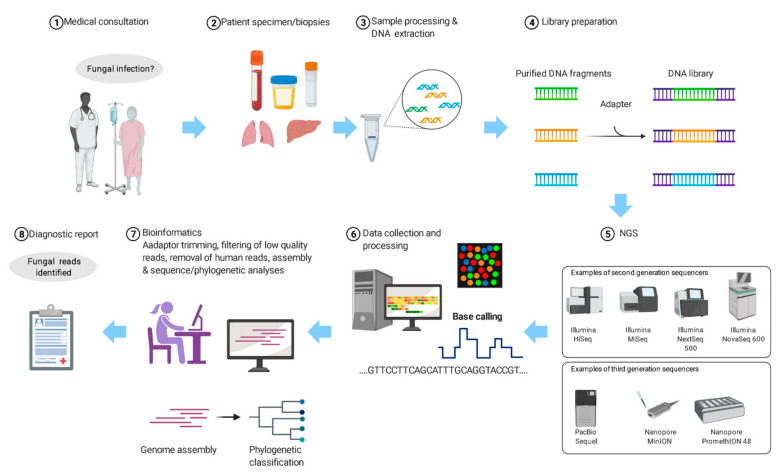
The workflow of using next-generation sequencing (NGS) for the diagnosis of suspected fungal infection.

Since using NGS for laboratory diagnosis is still expensive and labour-intensive and requires expertise, particularly the part on bioinformatics analysis, careful patient selection is crucial in order to generate the highest yield and make it most cost-effective (Figure 1). Patients who are most suitable for using NGS for laboratory diagnosis are those with culture-negative clinical syndromes, no matter whether it is pyrexia of unknown origin or the clinical features have already been pointing to a specific organ-system, such as meningitis. Preliminary investigations, including cultures, various serological tests and even molecular tests have been carried out, but there is still no idea regarding what the patient is suffering from. Before the use of NGS, clinical specimens may be subject to pan-bacterial, pan-fungal PCR, etc. With NGS in place, the samples, usually sterile body specimens such as blood, cerebrospinal fluid, peritoneal fluid, etc., can be subject to pan-sequencing.

The exact procedures of sample processing depend on the NGS platform used. In a nutshell, DNA is extracted from the sample and a library is prepared (Figure 1). The library is then subject to NGS. The NGS platforms commonly used for microbiology diagnosis are Illumina’s MiSeq System, Life Technologies’ Ion Personal Genome Machine (PGM) System, BGI’s BGISEQ-50/500 (now upgraded and rebranded as MGI’s DNBSEQ-G50/G400) Systems and Oxford Nanopore Technologies MinION System. The sequencing data will be processed using software that trims the adaptor, filters the low-quality reads and removes the human reads. The processed sequences will be assembled if necessary and subject to robust bioinformatics analysis. The number of reads that match different fungal species and other microorganisms will be generated.

## 3. Laboratory Diagnosis of Fungal Infections by NGS

A literature search of the PubMed database performed on 1 June 2021 using the keywords ‘(“fungal infection” OR “fungal infections” OR “fungal disease” OR “fungal diseases” OR mycosis) AND (“next generation sequencing” OR NGS OR metagenomics OR mNGS OR cfDNA OR mcfDNA)’ retrieved 265 articles. Of these, 42 were included in this review after manual examination as they represented case reports, series, or cohorts, while the remaining publications were excluded since they were considered out-of-scope, consisting of review articles, evaluation studies, or articles that lacked clinical information. A second search strategy using the keywords ‘(fungal infection OR mycosis) AND (next generation sequencing OR NGS OR mNGS OR metagenomics)’ retrieved 478 articles. After the removal of duplicated results and further manual examination, five additional articles were included in this review. Lastly, publications were searched using specific fungal names and after manual examination five extra articles were added. Therefore, in total 52 articles were included in this review [26,27,28,29,30,31,32,33,34,35,36,37,38,39,40,41,42,43,44,45,46,47,48,49,50,51,52,53,54,55,56,57,58,59,60,61,62,63,64,65,66,67,68,69,70,71,72,73,74,75,76,77].

**Figure 2:**
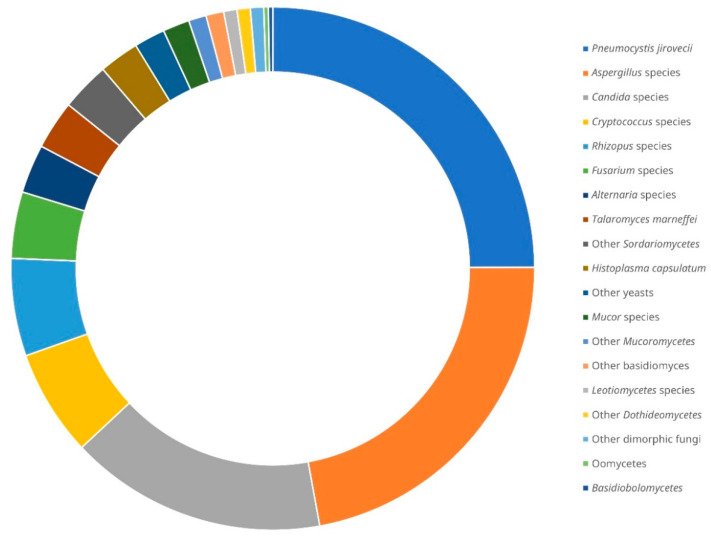
Distribution of fungal species detected by next-generation sequencing from clinical samples of patients with suspected infections reported in the literature [26,27,28,29,30,31,32,33,34,35,36,37,38,39,40,41,42,43,44,45,46,47,48,49,50,51,52,53,54,55,56,57,58,59,60,61,62,63,64,65,66,67,68,69,70,71,72,73,74,75,76,77]. There was a total of 319 cases with 365 fungi detected.

The first case of fungal infection diagnosed by NGS was reported in 2014. It was a case of allergic bronchopulmonary mycosis where *A. fumigatus* and *Schizophyllum commune* sequences were detected in the homogenised sputum and mucoid plugs of the patient [26]. Since then, up to the time of writing, a total of 319 cases of fungal infections diagnosed by NGS were described in the literature, with some in case reports and others in case series or cohorts [26,27,28,29,30,31,32,33,34,35,36,37,38,39,40,41,42,43,44,45,46,47,48,49,50,51,52,53,54,55,56,57,58,59,60,61,62,63,64,65,66,67,68,69,70,71,72,73,74,75,76,77]. In 40 out of the 319 cases (12.5%), more than 1 fungus was detected by NGS [26,27,28,32,34,39,41,44,45,53,59,62,64,67,73,74]. *Pneumocystis jirovecii* is the predominant fungus reported (~25% of the fungi detected), followed by *Aspergillus* species (~22%), *Candida* species (~16%), *Cryptococcus* species (~7%), *Rhizopus* species (~6%), *Fusarium* species (~4%), *Alternaria* species, *Talaromyces marneffei* and other *Sordariomycetes* (~3% each), *Histoplasma capsulatum*, other yeasts and *Mucor* species (~2% each), as well as some other rare fungal species (collectively ~5%) (Figure 2). Excluding *P. jirovecii,* which is generally unculturable, out of the remaining 274 fungi detected by NGS, only 105 (~38%) were recovered by fungal culture from the same or different specimens from the respective patients [26,29,30,31,32,33,34,35,36,37,38,39,43,45,46,49,52,54,57,59,62,64,66,67,71,73,76,77].

The majority of *P. jirovecii* infections diagnosed by NGS were reported in case series [34,36,39,41,48,53,55,58,62,64,65,67,72,75]. All the patients unanimously suffered from pneumonia. Although almost all patients were immunocompromised, HIV infection was very rare. This is very different from the general epidemiology of *P. jirovecii* infections, of which HIV infection is the most important risk factor. In fact, only 1 out of the 91 patients were HIV-positive [72]. The most common reasons for immunosuppression were haematological malignancies on chemotherapy and autoimmune diseases or renal transplant recipients on corticosteroid and/or other immunosuppressive treatment. This is because for HIV-positive patients with *P. jirovecii* infections, the fungal loads in their respiratory tracts are usually high and direct silver methenamine staining, sometimes even using induced sputum samples, is often sufficient for making a diagnosis. In contrast, for the other immunocompromised patients, the fungal load is usually low and bronchoscopic examination has to be performed to collect samples that give higher yield. For the cases of *P. jirovecii* pneumonia diagnosed by NGS, less than 20% were *P. jirovecii* positive by microscopic examination of the patients’ bronchoalveolar lavage or sputum samples after silver methenamine or immunofluorescence staining [34,39,53,58,62,64,65,67].

In addition to *P. jirovecii*, the epidemiology of the 11 cases of *T. marneffei* infections diagnosed by NGS are also different from talaromycosis in other patients (Table 1) [33,38,42,45,49,59,66,68,73,74,77]. *T. marneffei* is a thermally dimorphic fungus endemic in Southeast Asia. In some cases, the fungus is difficult to grow and no commercial serological test is available. Traditionally, most patients who suffer from talaromycosis are HIV-positive. However, in recent years, there is an emergence of *T. marneffei* infections in HIV-negative patients who have either primary or secondary immunodeficiencies [78]. Among the 11 patients with talaromycosis diagnosed by NGS, 5 were clinically diagnosed as tuberculosis (disseminated tuberculosis in case 1 [33], tuberculous meningitis in case 3 [42], pulmonary tuberculosis in cases 7 [66] and 9 [73] and tuberculosis peritonitis in case 10 [74]). Empirical anti-tuberculosis therapy was given in these five patients and NGS was performed because they did not respond to or deteriorated while on the treatment for tuberculosis. Another two patients were clinically diagnosed to have tumour (lymphoma in case 6 [59] and iris tumour in case 8 [68]). More than 60% of the 11 patients were HIV-negative [33,38,42,49,59,66,73,77], most likely the main reason why *T. marneffei* was not suspected in the first place. When talaromycosis was confirmed in a HIV-negative patient and no other obvious immunocompromised status was found, other primary immunodeficiency conditions, such as presence of anti-interferon-gamma autoantibody, hyper-IgE syndrome and hyper-IgM syndrome, should be looked for [78]. For example, genetic testing was performed in case 5 because the patient was HIV-negative and she had a history of recurrent infections in her childhood and loss-of-function *STAT3* mutation was discovered [49]. Similarly, *TSC2* mutation was detected in the patient in case 11 [77]. It is notable that patients with *T. marneffei* infections can be tested positive by the serum galactomannan assay [79], as shown in 2 of the 11 patients (cases 9 [73] and 10 [74]), because the immunoreactive β-galactofuransyl side chain of *Aspergillus* galactomannan [80] is also present in the cell wall of *Talaromyces* species of section *Talaromyces* [81], which encompasses *T. marneffei* [82].

Although detection of *P. jirovecii* or *T. marneffei* usually means that it is the culprit of the infection, the presence of *Aspergillus*, *Candida* or some other fungal sequences in clinical samples have to be interpreted with great care, as they sometimes may just represent colonisers or even contaminants. In a case series of prosthetic joint infections where various fungi were detected by NGS, *A. niger* sequences were detected in the intraoperative synovial fluid and prosthetic sonicated fluid from one of the patients with right hip prosthesis [57]. However, *Staphylococcus epidermidis*, a typical pathogen of prosthetic joint infection, was also found in these samples; and, in fact *S. epidermidis*, but not *Aspergillus*, was isolated from the intraoperative specimen. Moreover, fluconazole, which the *A. niger* should be resistant to, was given for the treatment of the infection; but the infection was cured. These suggested that the *A. niger* sequences may just be contaminants in this patient. In another case of pseudotumoral infection of the ascending colon that required hemicolectomy, Periodic acid–Schiff-stained histological section showed large hyphae surrounded by a thick eosinophic material, typical of *Basidiobolus* infection [28]. ITS amplification followed by NGS using the colon tissue revealed that 80% of the sequences matched *Basidiobolus meristosporus*, whereas the remaining were from *Malassezia globosa*, *Malassezia restricta,* and *Candida zeylanoides* [28]. In such circumstances, it is likely that the *Malassezia* and *Candida* sequences are contaminants or these fungi may be colonising the colonic mucosa. Incorrectly identifying the causative agent for an infection would pose a major risk for over-treatment to the patient with potentially toxic drugs or harmful procedures, which are entirely not necessary, and/or for physicians that are stopping to look for the actual reason for the patient’s deterioration and managing it. Training should be provided to physicians to update them regarding this emerging NGS technology for laboratory diagnosis of infection and clinical microbiologists should also be consulted when interpreting NGS results. Although there is currently no clinical trial to test and/or validate the performance of NGS for infectious disease diagnosis, this does not hinder the use of NGS for helping diagnose suspected infections when initial microbiological investigations do not yield anything fruitful and conclusive.

## 4. Opportunities, Obstacles, and Future Trend

The most important and attractive advantage of using NGS for laboratory diagnosis is that it can pick up all pathogens. Since it sequences all DNA present in the clinical sample, theoretically it will be able to detect bacteria, fungi, DNA viruses, and parasites. If RNA viruses are suspected, an additional step of reverse transcription can be used. When a physician faces a patient with pyrexia of unknown origin or other culture-negative infectious disease syndromes, he/she would welcome such a laboratory test that can pick up everything, including those difficult-to-grow, rarely occurred, or even previously unreported pathogens that the physician has not suspected, although this is against the traditional approach of patient care where the clinician has to suspect a diagnosis and order the specific laboratory test to confirm it. For example, as we described above, *T. marneffei* infection is not commonly on the list for HIV-negative patients in the mind of most clinicians, but it is repeatedly diagnosed using NGS. Somehow, NGS is analogous to a positive emission tomographic scan in radiology, which is able to locate the organ/system in patients with pyrexia of unknown origin.

In addition to being capable of detecting a wide range of pathogens, this one-technology-for-all-pathogen laboratory test can also enable us to do away with the trouble of designing and maintaining multiple sets of PCR reactions for these difficult cases in the long run. It is almost impossible to have PCR reactions that can detect all microorganisms, as different sets of primers have to be designed for different groups of pathogens. Moreover, different PCR reactions have different optimal conditions, which require a lot of expertise and experience to set up and maintain a whole set of PCR tests for such a high diversity of pathogens, even in reference laboratories. With the use of NGS, which does not require specific, conserved, or degenerate primers for different purposes, such a cumbersome set up would be unnecessary. Furthermore, although pan-bacterial and pan-fungal PCR using primers targeting the 16S rRNA gene and ITS can amplify novel bacteria and fungi, respectively, such primers are not available for viruses, as different families of viruses do not possess conserved sequences as in bacteria and fungi. Such a problem can be overcome by NGS when a previously undescribed microbe is the culprit in a particular patient.

Similar to other molecular technologies, NGS cannot fully replace phenotypic methods for detection of antifungal resistance, yet it can be used for rapid resistance detection in specific scenarios. Measurement of the growth of a fungal isolate in the presence of antifungal agents is the gold standard for examining antifungal resistance. This is because resistance can be resulted from various mechanisms, including previously undescribed mechanisms or mutations. As phenotypic methods are often tedious to perform and slow to get results, molecular methods such as PCR–sequencing have been used for rapid detection of antifungal resistance in situations where resistance is a result of relatively homogenous mutations. As NGS is able to pick up known resistance mutations, in theory it will be useful for the rapid detection of certain well-reported antifungal resistance mechanisms in some fungi that we currently use PCR–sequencing to detect. However, this relies on high sequencing accuracy, which in turn depends on high sequencing coverage of the mutation site.

At the current stage, the main obstacle to widespread use of NGS for laboratory diagnosis of fungal infections is still the high cost of NGS. A few years ago, NGS required expensive equipment, such as Illumina’s HiSeq System, Life Technologies’ SOLiD System, and PacBio’s RS/Sequel Systems, constituting a substantial investment (i.e., machine costs and the necessary infrastructure) that can cost up to USD 10 million. With the advent of benchtop NGS instrumentation, some newer models of sequencers, such as Illumina’s MiSeq System and Life Technologies’ PGM System, are able to deliver high-throughput sequencing on bench, which consume less laboratory space and are less expensive than the larger NGS platforms. In order to make more efficient use of the sequencing capacity of the NGS platforms, it will be more economical if multiple specimens are sequenced in a single run; i.e., the sequencing data generated by each platform are generally higher in quantities than required per specimen. With this strategy, the cost of sequencing one sample is around USD 500 [83]. In 2016, Oxford Nanopore Technologies has introduced its first sequencing device, MinION, into the market. It is a pocket-sized sequencing device of <450 g and costs around USD 1000, offering a portable and affordable alternative to the conventional NGS sequencers. Despite such a reduction of the investment cost on the hardware, the cost for sequencing one sample using the MinION device is still around USD 1500 (machine cost inclusive), which is still not affordable for most healthcare systems. In recent years, commercial services for NGS-based pathogen detection such as the Karius Test, whose pathogen list includes over 400 fungi, have emerged, which makes the costs for testing individual samples more favourable. This cell-free DNA test was developed based on blood samples from patients with invasive infections for pathogen detection, and it has been successfully applied for the diagnosis of fungal infection in several cases [36,47,69].

When the cost of NGS is further reduced, expertise becomes more widely available (such as technicians for library construction and bioinformaticians for the analysis of raw NGS data, etc.), and other obstacles are overcome, the routine use of NGS for laboratory diagnosis of infectious diseases would rely on computational capacity. Irrespective of the methodology used for laboratory diagnosis, it would involve extracting data from a sample and match the data collected to a database. The traditional way of identifying filamentous fungi involves extracting morphological data and comparing it with images in a textbook. For other methods, such as biochemical tests, matrix-assisted laser desorption/ionization–time-of-flight mass spectrometry (MALDI–TOF MS) as well as Sanger DNA sequencing of the ITS and/or other conserved regions, involve collecting biochemical profiles, protein mass spectra, and DNA sequences and comparing them with the corresponding databases using computer algorithms. For these methods, the file sizes of the databases are usually relatively small and the memories needed to compute data comparison are in general not demanding. Therefore, such databases can be downloaded/installed locally in the users’ computers for computation, as in the case for MALDI–TOF MS data analysis; or they can be loaded on web servers with a graphic user interface where users can upload their own data to the internet and have data comparison performed online, as in the case for the analysis of Sanger DNA sequences using Web BLAST. On the other hand, the analysis of NGS data for pathogen identification is much more computationally challenging. This is because the file size of the entire nucleotide collection (nr/nt) from the International Nucleotide Sequence Database Collaboration (INSDC) is over 100 Gb (as of 12 April 2021) and a much larger memory size is necessary to perform sequence searches locally. There is also currently no web server designated for this purpose available since a tremendously huge memory would be essential to allow concurrent data analysis by multiples of users from all over the world. Such a high reliability on computational power has never been seen in other laboratory diagnostic methods; and therefore, the day-to-day use of NGS for more routine laboratory diagnosis would rely on further improvement in data processing capabilities both locally and online. At present, to avoid this problem the application of this technology for pathogen detection/identification usually only involves the comparison of NGS data with pre-selected sub-datasets from the INSDC. Such data filtering greatly reduces the file size of the database and eases local data analysis. For example, only genomes of microbial pathogens are downloaded as references for the metagenomic NGS approach. However, because only a limited number of microbial, especially fungal, genomes are available at the time being, microbial pathogens without draft/complete genomes, thus not included for comparison, will be missed for detection/identification. Alternatively, if only curated sequences of DNA barcodes (e.g., ITS, 28S nrDNA and 18S nrDNA for fungi) are retrieved and used as reference data, since these DNA barcodes only constitute a very small portion of the genetic materials that microorganisms carry, the majority of the metagenomic NGS data will then go unmapped.

## 5. Concluding Remarks

The proof of concept that NGS is useful for the diagnosis of fungal infections has been demonstrated and shown to improve individual patient management. The number of cases reported every year has been increasing in the last eight years, indicating that it has become more popular. At the moment, the main obstacle for its more routine use is still the high cost. When the cost of NGS is further reduced, expertise becomes more widely available, and other obstacles are overcome, NGS would be a useful tool in the armamentarium for laboratory diagnosis of fungal infections, particularly for difficult-to-grow fungi and cases with low fungal loads. We also expect that NGS would help discover novel fungal pathogens, especially those that cannot be readily isolated.

## Figures and Tables

**Table 1:** Reported cases of talaromycosis diagnosed with the aid of next-generation sequencing.

Case	Sex/Age (Years) ^1^	Underlying/Predisposing Medical Conditions ^2^	Clinical Syndrome	Fungal Culture ^3^	Other Conventional Diagnostics for Fungal Infections ^4^	NGS Strategy and Platform ^5^	Fungal Species Detected by NGS ^6^	Antifungal Treatment ^7^	Outcome	Reference
1	M/22	None (HIV-negative)	Disseminated infection	Blood, sputum, CSF and BAL fluid: negative; skin lesion: *T. marneffei* 3 d post-1st NGS	Blood and CSF cryptococcal antigen test: negative	mNGS by BGISEQ-100	BM: *T. marneffei* (126 reads); skin lesion: *T. marneffei* (967 reads); BAL fluid: *T. marneffei* (172 reads); CSF: *T. marneffei* (120 reads)	dAmB and ITZ	Survived	[33]
2	M/23	None	Disseminated infection	BM: negative, positive 14 d post-NGS	Not mentioned	mNGS, platform not mentioned	BM: *T. marneffei* (3 reads); blood: *T. marneffei* (1 read)	Not mentioned	Survived	[38]
3	F/33	None (HIV- and autoimmune disease-negative)	Subacute meningitis	Sputum, blood, BM and CSF: negative	Histopathology on sputum, blood, BM and CSF smears: negative; G and GM tests: negative	mNGS, platform not mentioned (by BGI)	CSF: *T. marneffei* (1st: 49 reads; 2nd: 6 reads)	VRZ	Survived	[42]
4	M/52	HIV-positive	Disseminated infection	Sputum: negative; blood: *T. marneffei*	G and GM tests: negative; histopathology on lymph node biopsy: positive; PCR–ITS sequencing on lymph node biopsy: *T. marneffei*	mNGS, platform not mentioned	Lymph node biopsy: *T. marneffei* (4001 reads), other fungi (363 reads)	AmB and VRZ	Survived	[45]
5	F/34	*STAT3*-mutation	Chronic pneumonia syndrome	Sputum and blood: negative; BAL fluid and lung biopsy: *T. marneffei*	Plasma and BAL fluid GM and cryptococcal antigen test: negative	mNGS, platform not mentioned	BAL fluid: *T. marneffei* (566 reads)	ITZ	Survived	[49]
6	M/24	None (HIV-negative)	Disseminated infection	Blood: *T. marneffei*	Histopathology on lymph node biopsy: positive	mNGS, platform not mentioned	*T. marneffei* (~9400 reads), *Cladosporium sphaerospermum* (~97 reads), *Melampsora pinitorqua* (~97 reads) and *Plasmopara halstedii* (~97 reads)	AmB and VRZ	Deceased due to acute liver failure	[59]
7	M/29	None	Chronic pneumonia syndrome	BAL fluid and transbronchial biopsy: positive with no ID, identified as *T. marneffei* post-NGS	None	mNGS, platform not mentioned (by BGI)	BAL fluid: *T. marneffei* (3207 reads)	Pre-NGS: ITZ Post-NGS: ITZ	Survived	[66]
8	M/25	Syphilis, HIV-positive, recovered TB	Iris tumour, skin lesions	Blood: negative	CSF *Cryptococcus* test: negative; Gram staining and calcofluor white staining on skin lesion biopsy: negative; blood and aqueous humour GM test: negative; 26S rDNA PCR on aqueous humour: positive	mNGS by Ion Proton	Skin lesion biopsy: *T. marneffei* (93 reads); aqueous humour: *T. marneffei* (1435 reads)	VRZ and FCZ	Survived	[68]
9	M/79	DM (HIV-negative)	Chronic pneumonia syndrome	BM: negative; repeated sputa: *Candida albicans*; prolonged sputum: *T. marneffei* post-NGS	Sputum spear: fungal hyphae; serum GM test: positive	mNGS, platform not mentioned	BAL fluid: *T. marneffei* (38 reads) and *C. albicans* (5 reads); blood: *T. marneffei* (1 read)	Pre-NGS: CFG Post-NGS: VRZ	Survived	[73]
10	M/33	HIV-positive	Peritonitis	Blood and faeces: negative	GM test: positive; serum cryptococcal antigen: negative; BM smear: negative; histopathology on omentum majus biopsy: positive	mNGS, platform not mentioned (by BGI)	FFPE-omentum majus biopsy: *T. marneffei* (101,254 reads), *Wallemia mellicola* (1413 reads), *Aspergillus chevalieri* (195 reads)	Pre-NGS: AmB Post-NGS: AmB, ITZ	Survived	[74]
11	M/24	*TSC2*-mutation	Chronic pneumonia syndrome	Sputum and BAL fluid: negative; BAL fluid: positive 1 wk post-NGS	Cryptococcal capsular antigen and GM tests: negative; histopathological examination: fungal spores	mNGS, platform not mentioned	BAL fluid: *T. marneffei* (456 reads)	VRZ	Survived	[77]

^1^ F, female; M, male. ^2^ DM, diabetes mellitus; HIV, human immunodeficiency virus; *STAT3*, signal transducer and activator of transcription 3 gene; TB, tuberculosis; *TSC2*, tuberous sclerosis complex 2 gene. ^3^ BAL, bronchoalveolar lavage; CSF, cerebrospinal fluid; d, day; ID, identification; NGS, next-generation sequencing; wk, week. ^4^ BAL, bronchoalveolar lavage; BM, bone marrow; CSF, cerebrospinal fluid; G, glucan; GM, galactomannan; ITS, internal transcribed spacer; PCR, polymerase chain reaction; rDNA, ribosomal DNA. ^5^ BGI, Beijing Genome Institute; mNGS, metagenomic next-generation sequencing. ^6^ BAL, bronchoalveolar lavage; BM, bone marrow; CSF, cerebrospinal fluid; FFPE, formalin-fixed paraffin-embedded; NGS, next-generation sequencing. ^7^ AmB, amphotericin B; CFG, caspofungin; dAmB, amphotericin B deoxycholate; FCZ, fluconazole; ITZ, itraconazole; NGS, next-generation sequencing; VRZ, voriconazole.

## Data Availability

The data presented in this study are available on request from the corresponding authors. This study has also been presented during the Congress of the International Society for Human and Animal Mycology Asia Fungal Working Group (ISHAM Asia) 2021.
